# ABCG2 transporter inhibitor restores the sensitivity of triple negative breast cancer cells to aminolevulinic acid-mediated photodynamic therapy

**DOI:** 10.1038/srep13298

**Published:** 2015-08-18

**Authors:** Pratheeba Palasuberniam, Xue Yang, Daniel Kraus, Patrick Jones, Kenneth A. Myers, Bin Chen

**Affiliations:** 1 Department of Pharmaceutical Sciences, Philadelphia College of Pharmacy, University of the Sciences, Philadelphia, Pennsylvania, USA; 2 Department of Biological Sciences, Misher College of Arts & Sciences, University of the Sciences, Philadelphia, Pennsylvania, USA

## Abstract

Photosensitizer protoporphyrin IX (PpIX) fluorescence, intracellular localization and cell response to photodynamic therapy (PDT) were analyzed in MCF10A normal breast epithelial cells and a panel of human breast cancer cells including estrogen receptor (ER) positive, human epidermal growth factor receptor 2 (HER2) positive and triple negative breast cancer (TNBC) cells after treatment with PpIX precursor aminolevulinic acid (ALA). Although PpIX fluorescence was heterogeneous in different cells, TNBC cells showed significantly lower PpIX level than MCF10A and ER- or HER2-positive cells. PpIX fluorescence in TNBC cells also had much less mitochondrial localization than other cells. There was an inverse correlation between PpIX fluorescence and cell viability after PDT. Breast cancer cells with the highest PpIX fluorescence were the most sensitive to ALA-PDT and TNBC cells with the lowest PpIX level were resistant to PDT. Treatment of TNBC cells with ABCG2 transporter inhibitor Ko143 significantly increased ALA-PpIX fluorescence, enhanced PpIX mitochondrial accumulation and sensitized cancer cells to ALA-PDT. Ko143 treatment had little effect on PpIX production and ALA-PDT in normal and ER- or HER2-positive cells. These results demonstrate that enhanced ABCG2 activity renders TNBC cell resistance to ALA-PDT and inhibiting ABCG2 transporter is a promising approach for targeting TNBC with ALA-based modality.

Breast cancer is the most frequently diagnosed non-skin cancer and the second leading cause of cancer death in women^1^. Based on the expression of therapeutic markers, breast cancers are divided into three groups including estrogen receptor (ER) and/or progesterone receptor (PR) positive, human epidermal growth factor receptor 2 (HER2) positive, and triple-negative breast cancer (TNBC) that is lack of the expression of ER, PR and HER2^2^. Targeted therapies such as anti-hormone/hormone receptor and anti-HER2 treatments have greatly improved the treatment outcome of patients with ER- or HER2-positive tumors. However, there is no targeted therapy currently available for TNBC and chemotherapy remains the major therapeutic option for these patients. Despite substantial normal tissue toxicity, most TNBC patients do not respond to chemotherapy^3^. Thus, developing an effective and safe treatment for TNBC represents an urgent unmet medical need.

Photodynamic therapy (PDT) is a FDA-approved cancer treatment modality that uses photosensitizing chemicals (photosensitizers) to induce reactive oxygen species (ROS)-mediated tumor cell death upon laser light activation^4^. Preferential accumulation of photosensitizers in tumor tissues coupled with targeted delivery of activating light to tumor tissues ensures dual selectivity for tumor destruction. One PDT agent that exhibits excellent selectivity in some tumors is aminolevulinic acid (ALA)^5^. As a prodrug, ALA is metabolically converted to photosensitizer protoporphyrin IX (PpIX) in the heme biosynthetic pathway that occurs in almost all mammalian cells. However, compared with normal cells, tumor cells often show significantly higher ALA-mediated PpIX production likely due to alterations of heme biosynthetic enzymes in tumor cells^6^. Such a preferential PpIX production in tumor cells enables selective tumor destruction, particularly for skin cancers^7^. In addition to being a photosensitizer, PpIX is also a fluorophore. The fluorescent property of PpIX leads to the use of ALA as a tumor diagnostic agent and intraoperative tumor imaging probe during tumor surgery^8^.

Use of ALA for detecting and treating breast tumors is being actively explored^5^. Breast cancer cells show enhanced PpIX fluorescence than normal cells after ALA incubation^9^. ALA-based PpIX fluorescence imaging is effective in detecting early neoplastic and metastatic mammary tumors in transgenic mice^10^. PDT using ALA or its derivatives effectively inhibits breast cancer cell proliferation and tumor growth^11^,^12^. Its promise in diagnosing primary breast tumor as well as lymph node metastasis has been demonstrated in breast cancer patients, which shows that all primary tumors and metastatic lymph nodes examined in the study exhibit several-fold higher PpIX fluorescence than normal tissues after ALA administration^13^,^14^.

However, it is not yet known whether ER-positive, HER2-positive and TNBC cells have similar response to ALA-based imaging and therapy. To the best of our knowledge, there is no study comparing ALA-PpIX fluorescence and tumor cell response to ALA-PDT between different types of breast cancers. Such understanding has important clinical implications in using ALA-based modality for imaging and treating breast cancers. Through studying ALA-PpIX fluorescence, PpIX intracellular localization and cell response to ALA-PDT in a panel of human breast cancer cells including ER-positive, HER2-positive, TNBC cells, we found in the present study that TNBC cells had reduced ALA-PpIX fluorescence level and were resistant to ALA-PDT compared with ER- or HER2-positive cancer cells. Furthermore, our study demonstrated that inhibition of ATP-binding cassette transporter G2 (ABCG2) with Ko143 was able to reverse the resistance of TNBC to ALA-PDT by elevating PpIX level in mitochondria.

## Results

### TNBC cells exhibited lower ALA-PpIX fluorescence and less PpIX localization in mitochondria

**Figure 1:**
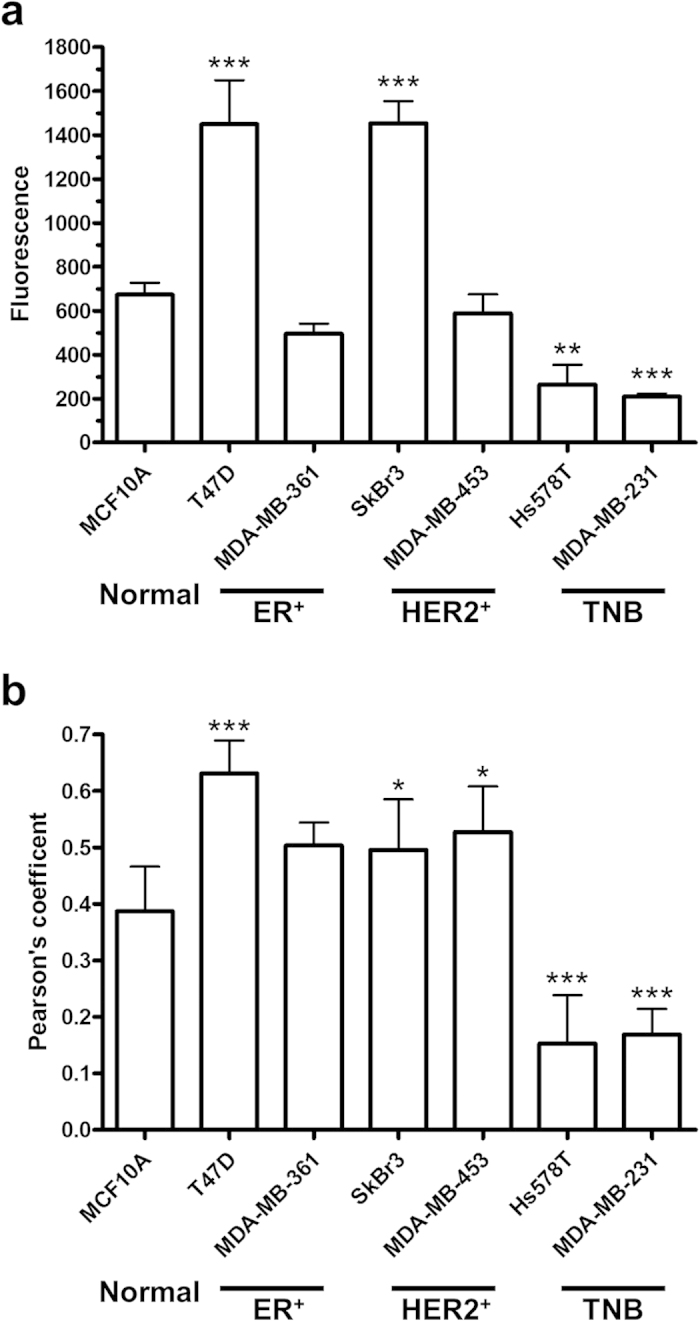
TNBC cells exhibited lower ALA-PpIX fluorescence and less PpIX localization in mitochondria than ER^+^ or HER2^+^ breast cancer cells. (**a**) ALA-PpIX fluorescence in normal and breast cancer cells. Cells were incubated with 1 mM ALA in complete medium for 4 h and fluorescence was measured by a flow cytometer in the FL3 channel. Data shown are results of three or four independent experiments. (**b**) Co-localization between ALA-PpIX fluorescence and mitochondrial marker in normal and breast cancer cells. Cells were incubated with ALA (1 mM for 4 h) and mitochondrial marker rhodamine 123 (250 ng/mL for 30 min) and imaged with a confocal fluorescence microscope. Pearson’s correlation coefficient between PpIX and corresponding rhodamine123 fluorescence was calculated using NIH ImageJ software (n = 4–9). All bars represent standard deviation (SD). *p < 0.05, **p < 0.01, ***p < 0.001, compared with MCF10A cells.

**Figure 2:**
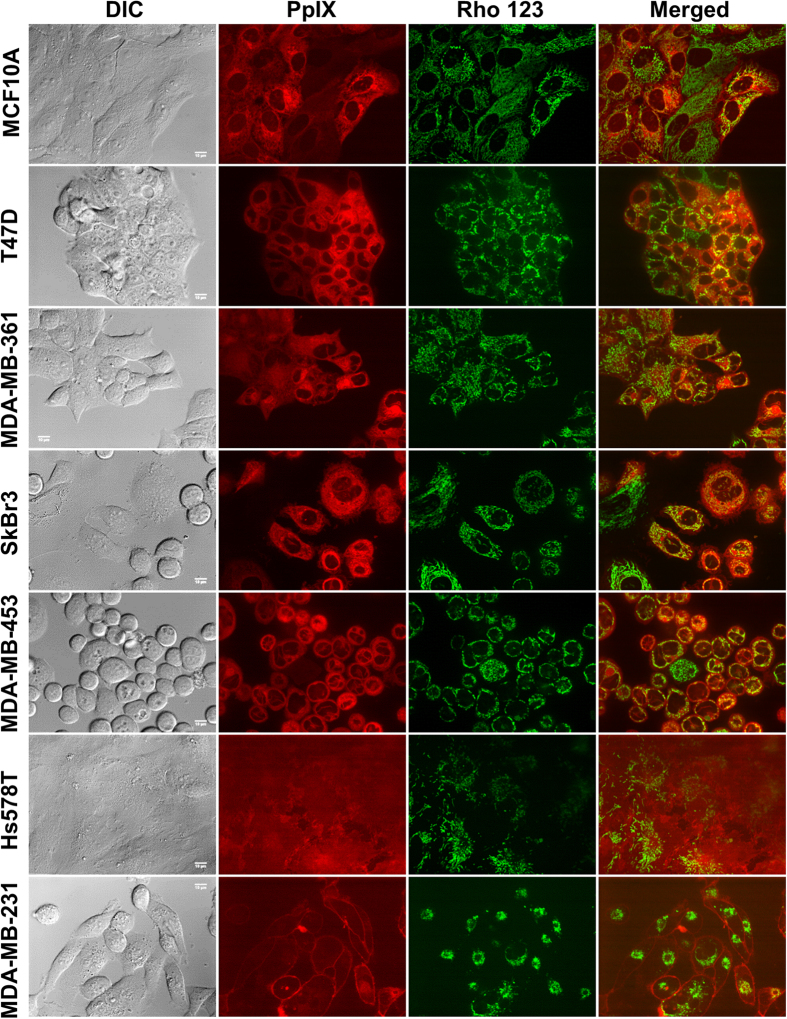
Confocal fluorescence images of ALA-PpIX and mitochondrial marker in normal and breast cancer cells. Cells were incubated with ALA (1 mM for 4 h) and mitochondrial marker rhodamine 123 (250 ng/mL for 30 min) and imaged with a confocal fluorescence microscope. Note that PpIX fluorescence in two TNBC cell lines was weaker and more localized to the cell membrane than other cell lines. Bars = 10 μm.

Heterogeneity in ALA-stimulated PpIX fluorescence was found in a panel of human breast cancer cells including ER positive (T47D, MDA-MB-361), HER2 positive (SkBr3, MDA-MB-453) and triple negative (Hs578T, MDA-MB-231) breast cancer cells (Fig. 1a). Particularly, T47D and SkBr3 cells showed significantly higher PpIX fluorescence than MCF10A normal breast epithelial cells (*p* < 0.001) whereas MDA-MB-361 and MDA-MB-453 cells exhibited similar fluorescence to MCF10A cells (*p* > 0.05). It is interesting to note that two TNBC cell lines had significantly lower PpIX fluorescence than MCF10A cells after ALA stimulation (*p* < 0.01). Co-localization analysis of PpIX fluorescence and mitochondrial marker in confocal images revealed a drastic difference in PpIX intracellular localization between TNBC and ER- or HER2-positive cancer cells (Fig. 1b). Compared with MCF10A cells, PpIX fluorescence in two TNBC cell lines had a significantly lower co-localization with mitochondria (*p* < 0.001) whereas significantly higher co-localization with mitochondria was found in ER-positive T47D and two HER2-positive cancer cell lines (*p* < 0.05). Although not statistically significant, PpIX fluorescence in ER-positive MDA-MB-361 cells showed a trend of increased mitochondrial localization compared with MCF10A cells. As shown in Fig. 2, PpIX fluorescence in ER- or HER2-positive cells overlaps well with the fluorescence of mitochondrial marker Rho123. In contrast, PpIX fluorescence in TNBC cells was reduced and much more diffuse. Substantial PpIX fluorescence was observed in the cell membrane in MDA-MB-231 cells.

### TNBC cells showed resistance to ALA-mediated PDT

**Figure 3:**
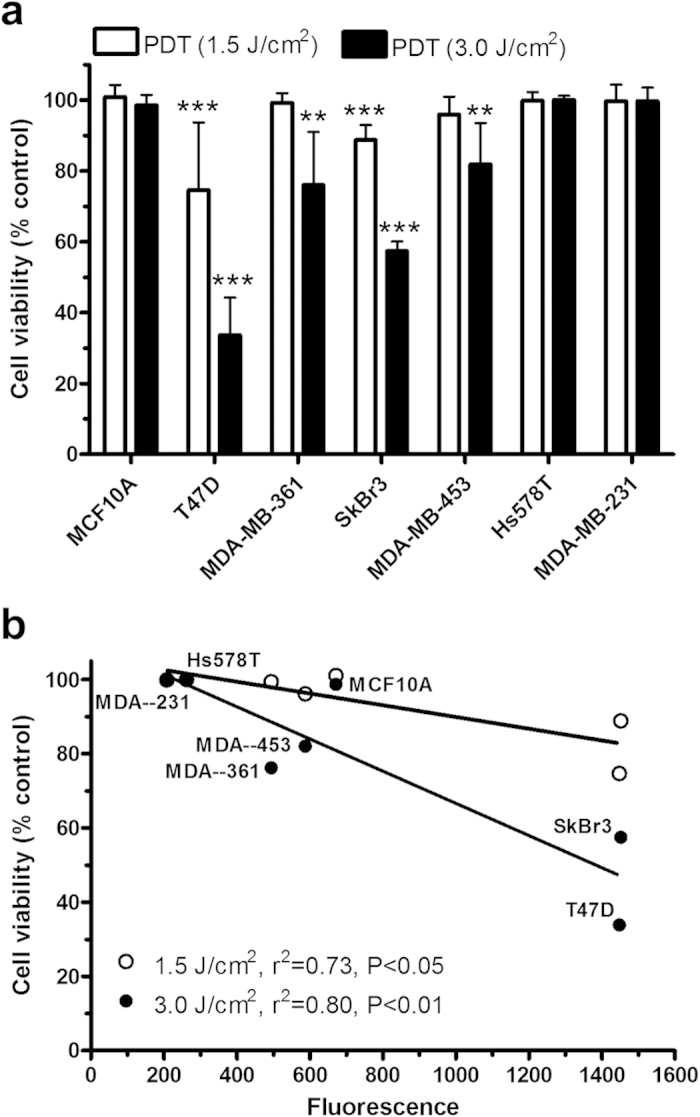
TNBC cells showed resistance to ALA-PDT. (**a**) Effects of ALA-PDT on cell viability. Cells were incubated with ALA (1 mM for 4 h) and then exposed to 1.5 (n = 10) or 3.0 (n = 6) J/cm^2^ light treatment. Cell viability was determined at 24 h after treatment. Bars represent SD. **p < 0.01, ***p < 0.001, compared with corresponding light only control which was normalized to 100%. (**b**) Inverse correlation between cell viability after ALA-PDT and PpIX fluorescence. Cell lines after 3.0 J/cm^2^ PDT treatment are labeled in the figure.

Cell viability after ALA-mediated PDT with two different light fluence (1.5 and 3.0 J/cm^2^) was shown in Fig. 3a. A dose-dependent decrease in cell viability was found in T47D and SkBr3 cells after PDT treatments. Although MDA-MB-361 and -453 cells showed no response to ALA-PDT with a lower fluence of 1.5 J/cm^2^, reduction in cell viability was seen after PDT with a higher fluence of 3.0 J/cm^2^. Similar to MCF10A cells, TNBC cells exhibited no response to ALA-PDT with either 1.5 or 3.0 J/cm^2^ light treatment. A significant inverse correlation was established between ALA-PpIX fluorescence and cell viability after ALA-PDT with either light dose (Fig. 3b). With the lowest ALA-PpIX fluorescence level, two TNBC cell lines showed no response to ALA-PDT, while T47D and SkBr3 cells with the highest PpIX fluorescence exhibited the highest sensitivity to ALA-PDT.

### ABCG2 inhibitor Ko143 enhanced ALA-PpIX fluorescence, reduced PpIX fluorescence heterogeneity and increased PpIX mitochondrial accumulation in TNBC cells

**Figure 4:**
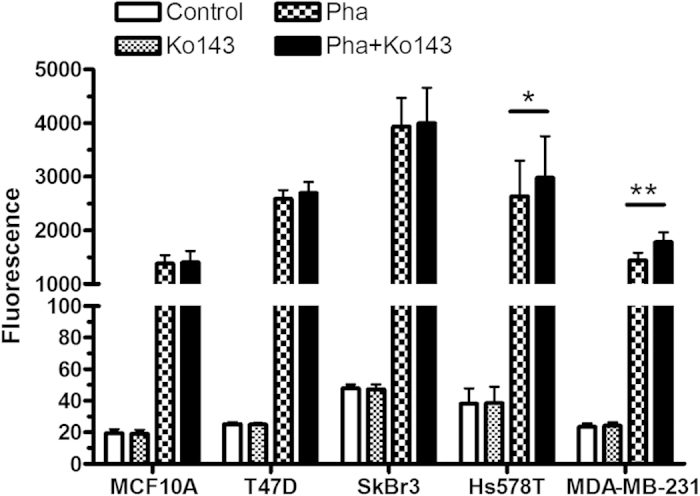
Assessment of ABCG2 transporter activity in normal and breast cancer cell lines. Cells were incubated with ABCG2 substrate pheophorbide a (Pha, 0.5 μM) alone, ABCG2 inhibitor Ko143 (1.0 μM) alone, and Pha (0.5 μM) combined with Ko143 (1.0 μM) for 1 h. Cell fluorescence was examined by a flow cytometer in the FL3 channel. Data shown are results of three to five independent experiments. Bars represent SD. *p < 0.05, **p < 0.01.

To compare the ABCG2 transporter activity between two TNBC cell lines resistant to ALA-PDT and PDT-sensitive breast cancer cell lines (T47D, SkBr3), cells were incubated with ABCG2 substrate pheophorbide a (Pha) with or without the presence of transporter inhibitor Ko143 and cell fluorescence of Pha was examined by flow cytometry. Treatment with Ko143 significantly increased Pha fluorescence in two TNBC cell lines (*p* < 0.05), but had no effect on Pha cellular uptake in MCF10A, T47D or SkBr3 cells (*p* *>* 0.05, Fig. 4), indicating that two TNBC cell lines exhibit enhanced ABCG2 transporter activity.

**Figure 5:**
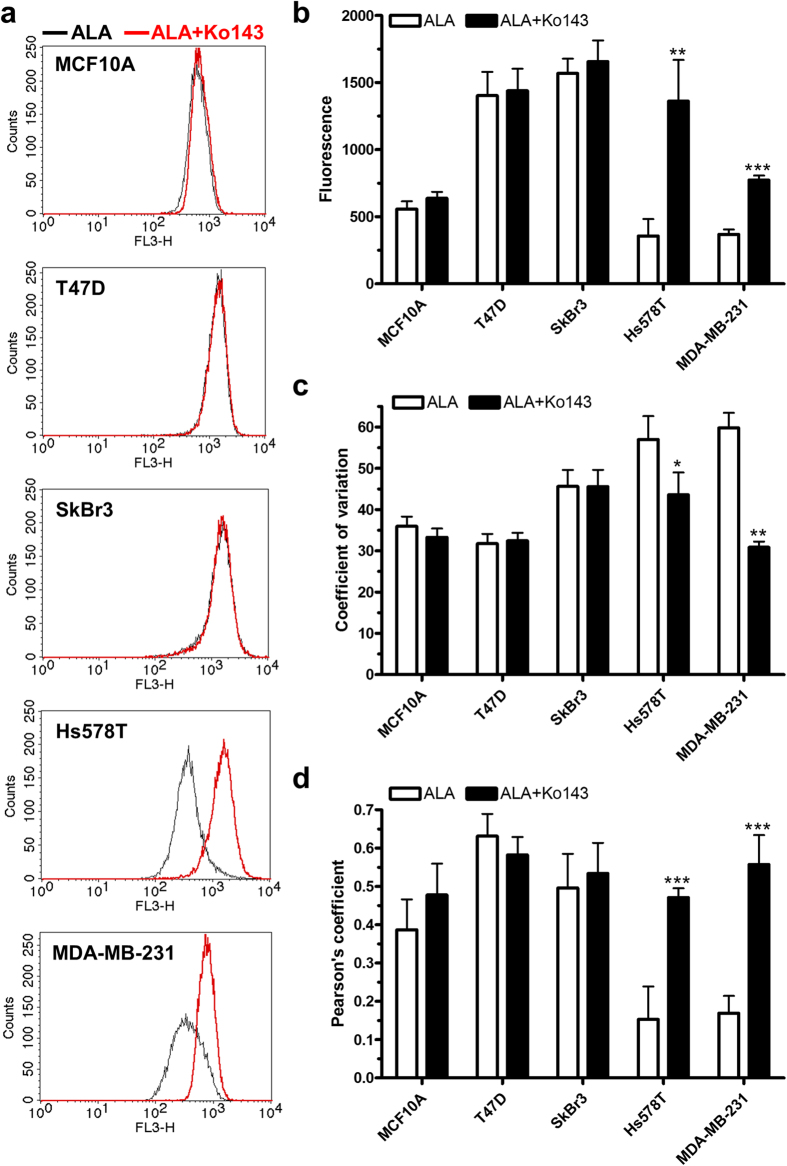
ABCG2 inhibitor Ko143 enhanced ALA-PpIX fluorescence, reduced PpIX fluorescence heterogeneity and increased PpIX mitochondrial accumulation in TNBC cells. (**a–c**) Cells were incubated with ALA (1 mM) alone or ALA (1 mM) combined with Ko143 (1 μM) in complete medium for 4 h and cell fluorescence was measured by a flow cytometer in the FL3 channel. (**a**) Representative histograms of ALA-PpIX fluorescence with or without Ko143. (**b**) Effects of Ko143 on the intensity of ALA-PpIX fluorescence. (**c**) Effects of Ko143 on the coefficient of variation (CV) of ALA-PpIX fluorescence. Data shown are results of three independent experiments. (**d**) Effects of Ko143 on ALA-PpIX mitochondrial localization. Cells were incubated with ALA (1 mM) alone or ALA (1 mM) combined with Ko143 (1 μM) in complete medium for 4 h and imaged with a confocal fluorescence microscope. To highlight mitochondria, cells were incubated with mitochondrial marker rhodamine 123 (250 ng/mL) for 30 min before confocal imaging. Co-localization between PpIX and corresponding rhodamine 123 fluorescence was determined by Pearson’s correlation coefficient (n = 4–9). All bars represent SD. *p < 0.05, **p < 0.01, ***p < 0.001, compared with corresponding ALA alone.

**Figure 6:**
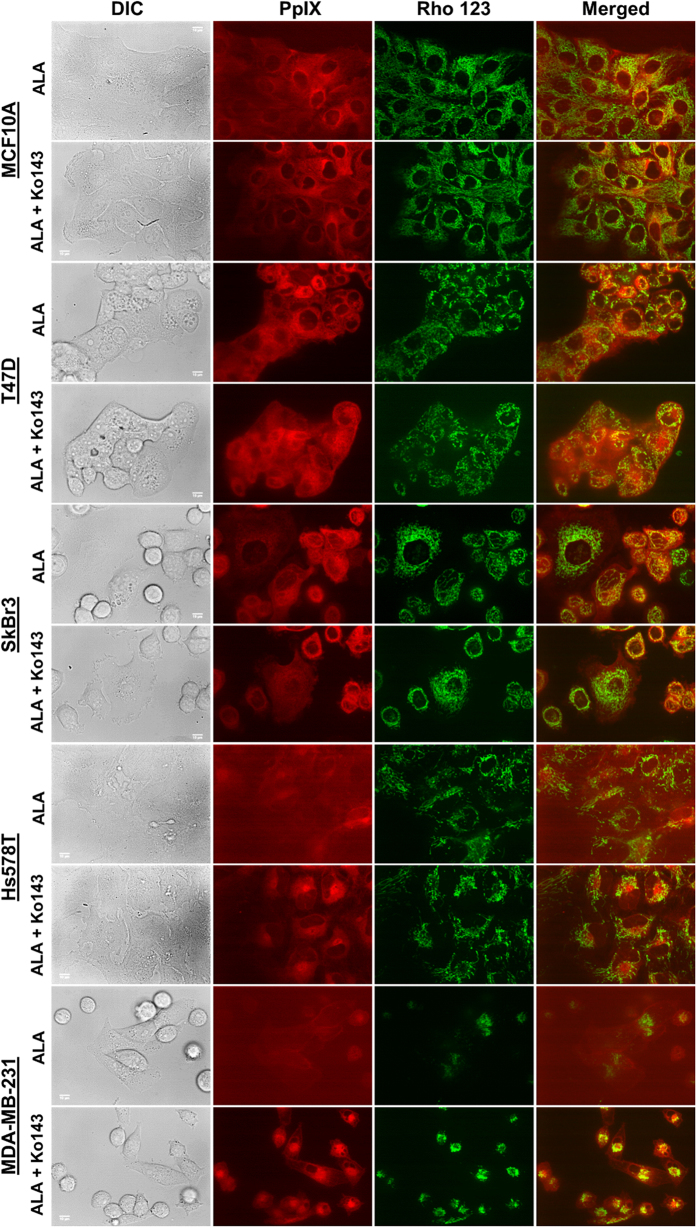
Confocal fluorescence images showing the effects of ABCG2 inhibitor Ko143 on ALA-PpIX fluorescence in normal and breast cancer cell lines. Cells were incubated with ALA (1 mM) alone or ALA (1 mM) combined with Ko143 (1 μM) in complete medium for 4 h and imaged with a confocal fluorescence microscope. To highlight mitochondria, cells were incubated with mitochondrial marker rhodamine 123 (250 ng/mL) for 30 min before confocal imaging. Note that Ko143 significantly increased PpIX fluorescence in mitochondria in two TNBC cell lines and had little effect on other cell lines. Bars = 10 μm.

Effects of Ko143 on ALA-PpIX fluorescence were examined by flow cytometry. Figure 5a shows ALA-PpIX fluorescence histograms of different cell lines treated with or without Ko143. Results of three independent experiments are shown in Fig. 5b. Ko143 (1 μM) had no significant effect on ALA-PpIX fluorescence in MCF10A, T47D and SkBr3 cells (*p* *>* 0.05). However, it significantly caused ALA-PpIX fluorescence increase in Hs578T (*p* < 0.01) and MDA-MB-231 (*p* < 0.001) cells by more than three and two times, respectively. Ko143 treatment was also found to significantly decrease coefficient of variation in two TNBC cell lines (Fig. 5c), suggesting reduced heterogeneity in PpIX fluorescence in these cells. Co-localization analysis of PpIX fluorescence and mitochondrial marker demonstrated that Ko143 significantly increased PpIX localization in mitochondria in Hs578T and MDA-MB-231 cells (*p* < 0.001, Fig. 5d). Confocal fluorescence imaging indicated that Ko143 treatment greatly increased ALA-PpIX fluorescence in mitochondria in Hs578T and MDA-MB-231 cells and had no visible effects on PpIX fluorescence in the rest cell lines (Fig. 6).

### ABCG2 inhibitor Ko143 sensitized TNBC cells to ALA-PDT

**Figure 7:**
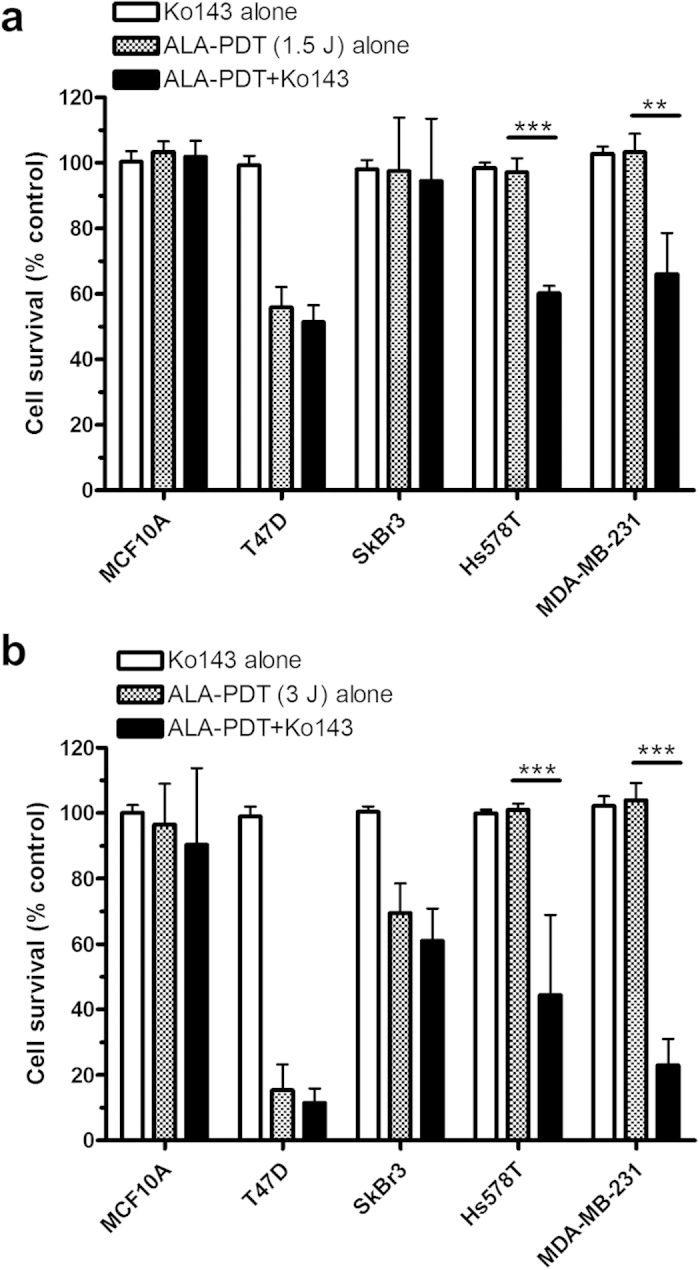
ABCG2 inhibitor Ko143 sensitized TNBC cells to ALA-PDT. (**a**) Cells were treated with Ko143 (1 μM) alone, ALA (1 mM for 4 h)-PDT with 1.5 J/cm^2^ fluence light alone and the combined treatments (n = 4). (**b**) Cells were treated with Ko143 (1 μM) alone, ALA (1 mM for 4 h)-PDT with 3.0 J/cm^2^ fluence light alone and the combined treatments (n = 6). Cell survival (relative to light only control) was examined at 24 h after treatments. All bars represent SD. **p < 0.01, ***p < 0.001, compared with corresponding ALA-PDT alone.

Effects of Ko143 on cell survival after ALA-PDT with two different light fluence (1.5 and 3.0 J/cm^2^) were shown in Fig. 7. Ko143 (1 μM) alone had no significant effect on cell survival (*p* *>* 0.05) in all cell lines examined. PDT treatment alone particularly at a higher light fluence reduced cell survival in T47D and SkBr3 cells, but was not effective in inhibiting the survival of Hs578T and MDA-MB-231 cells. Combination with Ko143 treatment significantly decreased cell survival in Hs578T and MDA-MB-231 cells treated with either 1.5 (Fig. 7a) or 3.0 (Fig. 7b) J/cm^2^ ALA-PDT and did not result in any significant effect on cell response to ALA-PDT in MCF10A, T47D or SkBr3 cells.

## Discussion

Through analyzing PpIX fluorescence in a panel of human breast cancer cells including ER-positive, HER2-positive and TNBC cells treated with PpIX precursor ALA, we observed a heterogeneity in PpIX fluorescence within and between different types of breast cancer cells. In the type of ER-positive cancer cells, PpIX fluorescence in T47D cells was more than 2-fold higher than in MCF10A normal breast epithelial cells while the other ER-positive MDA-MB-361 cells exhibited a similar PpIX fluorescence to the MCF10A cells (Fig. 1a). Similar variation was also seen in HER-2 positive cancer cells. PpIX fluorescence in SkBr3 cells was twice as much as in MCF10A cells and the other HER2-positive MDA-MB-453 cell line showed about the same fluorescence as MCF10A cells. However, both TNBC cell lines (Hs578T, MDA-MB-231) were found to exhibit PpIX fluorescence several times lower than MCF10A cells, suggesting a drastic difference from ER- or HER2-positive cells. As human breast cancer is known for its genetic and phenotypic diversity^15^, these results highlight a complex effect of diverse oncogenesis and tumor heterogeneity on PpIX fluorescence. More importantly, our data indicate that TNBC with reduced ALA-PpIX level will represent a challenge for ALA-based tumor detection and treatment.

Through analyzing PpIX intracellular localization, we found that ALA-PpIX in ER- or HER2-positive breast cancer cells tended to have higher mitochondrial localization than normal MCF10A cells whereas TNBC cells showed much less PpIX localization in mitochondria (Fig. 1b). As an endogenous photosensitizer biosynthesized inside mitochondria^16^, PpIX has been known to accumulate in mitochondria in different tumor cells and the mitochondrion is often considered as an important target of ALA-PDT for inducing tumor cell death^17^,^18^. With high PpIX fluorescence and substantial intracellular accumulation in mitochondria, T47D and SkBr3 cells exhibited good sensitivity to ALA-PDT (Fig. 3). Although MDA-MB-361 and MDA-MB-453 cells displayed similar PpIX fluorescence to MCF10A cells, PpIX tended to have higher mitochondrial localization in two cancer cell lines than in MCF10A cells. The finding that two cancer cell lines, but not MCF10A cells, were responsive to PDT demonstrates the importance of PpIX mitochondrial localization in determining cell response to ALA-PDT. Because of low PpIX fluorescence and reduced PpIX mitochondrial accumulation, two TNBC cell lines showed no response to ALA-PDT. The resistance of TNBC cells to ALA-PDT calls for an understanding of resistance mechanism and therapeutic approaches to sensitize TNBC cells to ALA-PDT.

The observation that PpIX fluorescence exhibited a diffuse and cell membrane bound pattern in the TNBC cells suggested to us a possibility of PpIX efflux transport. Because PpIX is known to be a substrate of ABCG2 transporter^19^,^20^, it is possible that enhanced ABCG2 transporter activity results in reduced PpIX in TNBC cells. ABCG2/breast cancer resistance protein (BCRP) is a member of ATP-binding cassette (ABC) transporter family that transports a variety of structure-unrelated endogenous and exogenous chemicals^21^. ABCG2 transporter is important for maintaining homeostasis by limiting the intracellular concentration of potentially toxic chemicals and inducing drug resistance by effluxing therapeutic agents. To determine whether TNBC cells possess enhanced ABCG2 transporter activity, we examined ABCG2 transporter activity in ALA-PDT-resistant TNBC cells and PDT-sensitive cells (T47D, SkBr3) using a specific ABCG2 transporter probe pheophorbide a (Pha) together with a selective transporter inhibitor Ko143^22^,^23^. We found that two TNBC cells exhibited higher ABCG2 activity than normal and PDT-sensitive cancer cells because incubation with Ko143 for 1 h significantly increased Pha cellular uptake in two TNBC cell lines and had no effect on other cell lines (Fig. 4). Inhibition of ABCG2 transporter activity with Ko143 significantly increased ALA-PpIX fluorescence in two TNBC cell lines (Fig. 5), demonstrating the involvement of enhanced ABCG2 transporter activity in reducing ALA-PpIX fluorescence in TNBC cells. Along with increased PpIX fluorescence, PpIX accumulation in mitochondria was also increased after Ko143 treatment (Figs 5 & 6), supporting a recent finding that ABCG2 transporter is localized in the mitochondria^24^. As a consequence of Ko143-induced increase in ALA-PpIX fluorescence and PpIX mitochondrial accumulation, both TNBC cell lines became sensitive to ALA-PDT even at a lower fluence (Fig. 7). These results demonstrate that enhanced ABCG2 transporter activity renders TNBC cell resistance to ALA-PDT by reducing PpIX cellular level, particularly in the mitochondria, which can be reversed by the inhibition of ABCG2 transporter activity.

It is interesting to note that Ko143 treatment significantly decreased PpIX fluorescence heterogeneity in two TNBC cell lines, especially in MDA-MB-231 cells. As intra-tumor heterogeneity in PpIX fluorescence is known to contribute to heterogeneous PDT response in clinic^25^, our results suggest the use of ABCG2 inhibitors to enhance PDT outcomes by decreasing the heterogeneity in PDT response. The finding that Ko143 was only able to enhance PpIX fluorescence and ALA-PDT response in two TNBC cell lines with increased ABCG2 transporter activity, but not in normal and other cancer cell lines without elevated ABCG2 activity, demonstrates the selectivity of this therapeutic enhancement. It also suggests that this approach will provide beneficial treatments only to tumors with enhanced ABCG2 activity. This notion is in agreement with previous studies using skin cancer cell lines where enhancement of ALA-PDT response by ABCG2 transporter inhibitors was only observed in ABCG2-positive cells but not in ABCG2-negative cells^26^,^27^,^28^.

Results from this study have important implications for using ALA-based modalities for imaging and treating breast cancers. Although not all ER- or HER2-positive tumors will exhibit increased ALA-PpIX fluorescence than normal cells, it appears that the majority, if not all, of these tumors will respond to ALA-PDT due to enhanced PpIX accumulation in mitochondria. However, for breast tumors with enhanced ABCG2 transporter activity, particularly TNBC where ABCG2 is often found expressed at a higher level than other types of breast tumors^29^, ALA-based modality needs to be combined with ABCG2 transporter inhibitors. We believe that, as primarily a local therapy, ALA-based modality is especially useful for breast tumor surgery, particularly in breast conserving tumor surgery that is being carried out at an increased rate. ALA-PpIX fluorescence can be used to highlight tumor tissues to guide tumor resection and ALA-PDT can be performed on the surgical site after tumor dissection to ensure complete inactivation of tumor cells. The feasibility of using ALA-based modality together with inhibition of ABCG2 transporter activity to facilitate complete removal of breast tumor tissues warrants further investigation.

In summary, we analyzed ALA-PpIX fluorescence, PpIX localization in mitochondria and cell response to ALA-PDT in normal human breast epithelial cells and different types of human breast cancer cells. For the first time, we reported that TNBC cells had reduced PpIX fluorescence, low PpIX mitochondrial accumulation and were resistant to ALA-PDT due to enhanced ABCG2 activity whereas ER-, or HER2-positive cells were responsive to ALA-PDT because of increased PpIX mitochondrial accumulation and/or increased PpIX fluorescence. ABCG2 transporter inhibitor Ko143 sensitized TNBC cells to ALA-PDT by increasing ALA-PpIX fluorescence, reducing PpIX fluorescence heterogeneity and enhancing PpIX mitochondrial accumulation while it had little effect on normal and other cancer cell lines. Results presented in this study call for the incorporation of ABCG2 transporter inhibitors into ALA-based modality for imaging and targeting TNBC that is currently an unmet medical need due to the lack of safe and effective therapeutic approaches.

## Methods

### Chemicals

Delta-aminolevulinic acid hydrochloride (ALA) from Frontier Scientific Inc. (Logan, UT) and rhodamine 123 from Life Technologies (Grand Island, NY) were dissolved in phosphate buffered saline (PBS) solution. Pheophorbide a (Pha) from Frontier Scientific Inc. and ABCG2 inhibitor Ko143 from Santa Cruz Biotechnology (Santa Cruz, CA) were dissolved in dimethyl sulfoxide (DMSO). All chemicals were sterilized through filters and stored in a −20 °C freezer.

### Cell culture

MCF10A human breast epithelial cells were maintained in Dulbecco’s Modified Eagle Medium (DMEM)/Ham’s F-12 (50/50) medium, supplemented with 5% horse serum (Atlanta Biologicals), insulin 10 μg/mL, epidermal growth factor (EGF) 20 ng/mL, cholera toxin 100 ng/mL, hydrocortisone 0.5 μg/mL and 1% antibiotics and antimycotics solution. Human breast cancer cell lines T47D and SkBr3 were cultured in RPMI 1640 and McCoy’s 5A respectively, supplemented with 9% fetal bovine serum (FBS, Atlanta Biologicals) and 1% antibiotics and antimycotics. Human breast cancer cell lines Hs-578T, MDA-MB-231, MDA-MB-453 and MDA-MB-361 were all cultured in DMEM medium supplemented with 9% FBS and 1% antibiotics and antimycotics. All cell lines were obtained from American Type Culture Collection (ATCC, Manassas, VA) and all cell culture media were obtained from Mediatech (Manassas, VA). Cells were maintained at 37 °C in a humidified incubator with 5% CO_2_.

### Flow cytometry analysis

Cellular PpIX or Pha fluorescence intensity was determined by flow cytometry. Cells were seeded in 60 mm cell culture dishes and grown for two days to reach about 70% confluence. Cell culture medium was removed and cells were incubated in complete medium containing 1 mM ALA for 4 h or 0.5 μM Pha for 1 h. For cells that needed treatments with ABCG2 inhibitor Ko143, Ko143 (1 μM) was added into medium together with ALA or Pha for treatments. After the incubation time, drug-containing medium was removed. Drug-treated as well as untreated control cells were rinsed twice with PBS, trypsinized and suspended in PBS. Cell suspensions were centrifuged and cell pellets were re-suspended in PBS for PpIX or Pha measurement. PpIX or Pha fluorescence was measured with a FACSCalibur flow cytometer (BD Biosciences) in the FL3 channel (488 nm excitation, 650 nm long-pass emission), which captured the second emission band of PpIX^30^. About 20,000 cells were measured and recorded for each experiment. Experiments were repeated at least three times.

### Confocal fluorescence microscopic imaging

Cells were implanted in glass bottom cell culture dishes (MatTek, Ashland, MA) and grown for two days. Cells were rinsed with PBS twice and incubated in complete medium containing ALA (1 mM) alone or ALA (1 mM) and Ko143 (1 μM) for 4 h. At about 30 min before due time, rhodamine 123 (250 ng/mL) was added into the medium for mitochondrial labeling. After drug containing medium was removed, cells were washed with PBS twice and incubated in serum free medium for confocal imaging.

Live-cell imaging was performed on a Nikon TiE (Eclipse) confocal microscope equipped with a CSU-X spinning disk confocal scan head (Yokogawa), a temperature-controlled linear encoded x, y robotic stage (ASI Technologies, Inc.), a multi-bandpass dichromatic mirror (Semrock) and bandpass filters (Chroma Technology Corp.) in a filter wheel. Microscope system was set to image the fluorescence of PpIX (405 nm excitation, 700 ± 37.5 nm emission) and rhodamine 123 (488 nm excitation, 525 ± 18 nm emission). Laser illumination was provided by a 50 mW monolithic laser combiner (MLC400, Agilent Technologies) and images were acquired using a Clara interline CCD camera (Andor Technology) through a 60 × (1.40 NA) oil immersion objective. The exposure time for PpIX and rhodamine 123 was set at 500 ms and 200 ms, respectively. Differential interference contrast (DIC) images were acquired using exposure times in the range of 100 to 200 ms at the same magnification. The microscope system, laser launch and image acquisition were controlled by Nikon Elements software.

Monochrome fluorescence images from different channels were pseudo-colored and merged to generate composite images. Co-localization between rhodamine 123 and PpIX fluorescence images was analyzed and represented by the Pearson’s coefficient with 1 indicating complete co-localization and 0 showing no co-localization. All images were processed and analyzed using NIH ImageJ software.

### PDT treatment & cytotoxicity assay

Cells were implanted in 96-well plates and grown for two days. For ALA-PDT, cells were incubated in complete medium with ALA (1 mM) or without ALA (for control) for 4 h before light treatment. For cells treated with ALA-PDT and Ko143, Ko143 (1 μM) was added together with ALA into the medium. After 4 h incubation, cells were treated with 5 mW/cm^2^ irradiance of 633-nm light for 5 min or 10 min, which results in light fluence of 1.5 and 3.0 J/cm^2^, respectively. Light illumination was provided by a diode laser system (High Power Devices Inc., North Brunswick, NJ), which is coupled to a 600-μm core diameter optical fiber fitted with a microlens at the end to achieve homogeneous irradiation. Light intensity was measured with an optical power meter (Thorlabs, Inc., North Newton, NJ). Immediately after light treatment, drug-containing medium was replaced with fresh complete medium and cells were returned to incubator for culture. Cell viability was determined at 24 h after treatment by CellTiter 96 Aqueous Non-Radioactive Cell Proliferation Assay (MTS assay, Promega, Madison, WI) following manufacturer’s instruction.

### Statistical analysis

Two-tailed student’s t-test was used to determine statistical significance between two groups. For multiple group comparison, one-way ANOVA test with Tukey’s post test was used. Statistical significance was accepted at *p* < 0.05.

## Additional Information

**How to cite this article**: Palasuberniam, P. *et al*. ABCG2 transporter inhibitor restores the sensitivity of triple negative breast cancer cells to aminolevulinic acid-mediated photodynamic therapy. *Sci. Rep*. **5**, 13298; doi: 10.1038/srep13298 (2015).
